# Predicting the Tumour Response to Radiation by Modelling the Five Rs of Radiotherapy Using PET Images

**DOI:** 10.3390/jimaging9060124

**Published:** 2023-06-20

**Authors:** Rihab Hami, Sena Apeke, Pascal Redou, Laurent Gaubert, Ludwig J. Dubois, Philippe Lambin, Dimitris Visvikis, Nicolas Boussion

**Affiliations:** 1 INSERM UMR 1101 “LaTIM”, CEDEX 3, 29238 Brest, France; 2 CERV, European Center for Virtual Reality, ENIB, CEDEX 3, 29238 Brest, France; 3 The D-Lab, Department of Precision Medicine, GROW-School for Oncology, Maastricht University, 6211 LK Maastricht, The Netherlands; 4 CHRU BREST, 29200 Brest, France

**Keywords:** radiotherapy, five Rs of radiobiology, tumour response, simulation, PET images

## Abstract

Despite the intensive use of radiotherapy in clinical practice, its effectiveness depends on several factors. Several studies showed that the tumour response to radiation differs from one patient to another. The non-uniform response of the tumour is mainly caused by multiple interactions between the tumour microenvironment and healthy cells. To understand these interactions, five major biologic concepts called the “5 Rs” have emerged. These concepts include reoxygenation, DNA damage repair, cell cycle redistribution, cellular radiosensitivity and cellular repopulation. In this study, we used a multi-scale model, which included the five Rs of radiotherapy, to predict the effects of radiation on tumour growth. In this model, the oxygen level was varied in both time and space. When radiotherapy was given, the sensitivity of cells depending on their location in the cell cycle was taken in account. This model also considered the repair of cells by giving a different probability of survival after radiation for tumour and normal cells. Here, we developed four fractionation protocol schemes. We used simulated and positron emission tomography (PET) imaging with the hypoxia tracer 18F-flortanidazole (18F-HX4) images as input data of our model. In addition, tumour control probability curves were simulated. The result showed the evolution of tumours and normal cells. The increase in the cell number after radiation was seen in both normal and malignant cells, which proves that repopulation was included in this model. The proposed model predicts the tumour response to radiation and forms the basis for a more patient-specific clinical tool where related biological data will be included.

## 1. Introduction

Cancer is considered the leading cause of death in many developed countries [1], and it is a major public health problem around the world [2]. In the future, this disease will become the leading cause of morbidity and mortality in all regions of the world [3]. Cancer is a family of diseases characterized by abnormal cell growth, which damages the cell’s DNA. Radiotherapy represents one of the main curative approaches and plays a central role in cancer therapy. Indeed, more than 50% of patients receive radiotherapy at some point during their treatment [4]. Radiotherapy aims to deliver enough radiation to kill tumour cells but as low as possible to limit damage to surrounding normal cells [5]. That is why fractionated radiotherapy, in most cases, is the common technique used to treat cancer with radiation [6]. Indeed, dividing the total dose into several smaller ones over a period of several days reduces the toxicity of healthy cells [4].

The effectiveness of radiotherapy depends on several factors and especially on the total dose delivered. However, the response of the tumour to a given treatment can vary from one patient to another. Several underlying biological effects that could explain the variation in the biological response of tissues occurring during fractionated radiotherapy have been identified. Such biological principles can be summarized by a simple viewpoint called the five Rs of radiobiology: repair, repopulation, reoxygenation, redistribution in the cell cycle and radiosensitivity [7].

The heterogeneity of the tumour response in patients with the same type of cancer and the non-uniformity of tumour cells within the same individual tumour remain a key challenge for the cure of cancer [8]. Understanding and predicting the response of the tumour and normal tissues to treatment is therefore essential to provide useful information on the effectiveness of radiotherapy. The development of a clinical decision-support system based on predictive and prognostic data factors could help clinicians in their decision-making [9].

In recent years, there has been a significant amount of interest in the search for an optimal and personalized treatment plan [10,11,12]. Many models have been developed to study tumour growth and the radiotherapy response to radiation [13]. Most of these studies were based on simulations using mathematical models to describe the processes determining the response of cells and tissues to radiation. For example, the model proposed by Lind et al. [14] is based on the interaction of two Poisson processes and took into account two distinct types of cell damage, potentially repairable and conditionally repairable.

The effects of repair and repopulation have been modelled [15]. In this work, population dynamics and the cell number after radiation were simulated by coupling the linear-quadratic (LQ) model with a repopulation model. Furthermore, the effects of damage repair, cellular repopulation and redistribution in the cell cycle have been modelled via an analytical model describing the effect of radiation on cell inactivation [16]. 

Fakir et al. [17] presented a mathematical niche model for estimating tumour control probability (TCP) characterizing the tumour repopulation due to the proliferation of cells that survive after irradiation. A recent study by Yang et al. [18] focused on the process of tumour repopulation during radiotherapy and its detection methods using 18F-FLT PET. Badri et al. [19] developed a stochastic radiation-scheduling concept and incorporated inter-patient variability. This approach was based on a linear-quadratic model, including tumour proliferation, while tumour repopulation was based on a simple exponential law starting after a “kick-off” time [20].

Several experimental studies have shown that tumour cells in a hypoxic environment have a higher probability of surviving after irradiation than cells in a well-oxygenated situation. Oxygen availability is closely linked with the micro-vascular network. In this context, Grogan et al. [21] presented a hybrid multiscale model to predict the cellular response to radiotherapy. They used a cellular automaton approach of tumour growth and a model for oxygen transport from blood vessels. Other models have been developed to study the dynamics of hypoxia and the effect of reoxygenation in fractionated radiotherapy [22,23,24,25,26]. In general, most simulation methods remain theoretical and are rarely compared with actual clinical data. In addition, to our knowledge, no study on the modelling of the tumour response to radiotherapy has taken into account these five processes together. Proposed methods are rarely confronted with the actual tumour microenvironment of a given patient. Furthermore, as stated above, hypoxia and the partial pressure of oxygen (pO_2_) [27] should be taken into account when the response to radiotherapy [28] is studied. Although several methods capable of simulating pO_2_ exist [29], the proposed approaches often neglect the dynamic aspects of oxygen pressure during treatment [30].

A mathematical model for the tumour response that included the cell cycle effect in the context of lung cancer was presented by Jeong et al. [24] for understanding the dynamics of tumour cells during radiotherapy. A series of recent studies also included the effects of the cell cycle [31,32]. Other analytical models focused on the impact of intrinsic radiosensitivity on the tumour cell population [33,34,35].

It appears from these studies that an ideal predictive model should integrate all known radiobiological processes [34] to take into account the personal biological parameters of each patient, especially at the beginning of treatment. In the present study, the main objective was to evaluate the potential of incorporating the five Rs of radiobiology in a simulation process. We developed an analytical model that included the effects of repair, repopulation, redistribution, reoxygenation and radiosensitivity on the tumour response to radiotherapy.

This new general approach is illustrated by the implementation of tumour [18F] HX4 PET images as input data. This tracer is a hydrophilic variant of the 2-nitroimidazole class of radiotracers, which is used as a marker of hypoxia for the preclinical evaluation and validation of PET imaging [36,37].

## 2. Materials and Methods

### 2.1. Model of Tumour Response to Radiotherapy

The proposed model was developed to describe the evolution and tumour response at both macroscopic and microscopic scales. In this work, the macroscopic scale at the tumour level is represented by PET images. These patient-dependent images are used as the input data, and at this scale, the model consists of a series of $N=N_{x}\times N_{y}\times N_{z}$ voxels, where $N_{x},N_{y}{,N}_{z}$ were the numbers of voxels in each direction for the PET image.

**Figure 1:**
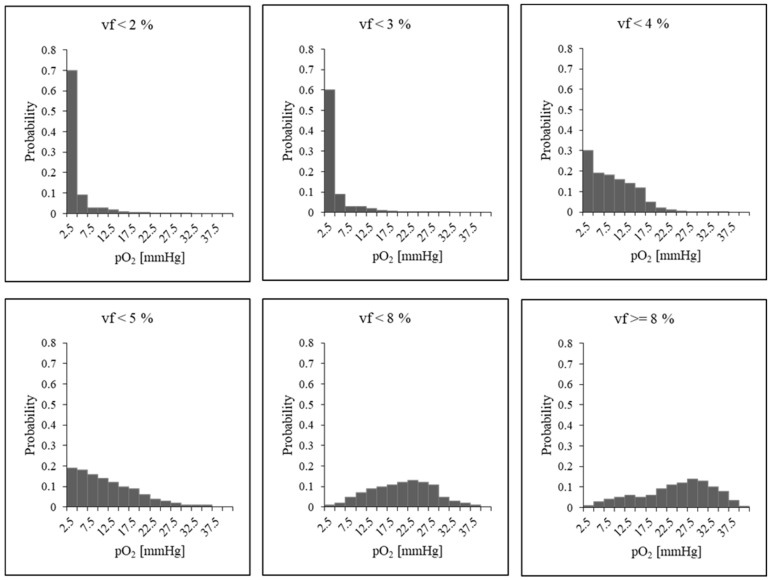
Simulated oxygen pressure (*pO*_2_) histograms for different vascular fraction values vf (upper: <1%, <3%, <4%; lower: <5%, <8%, ≥8%).

The total number of cells inside a voxel was constant, according to a fixed cell density *µ* (Table 1). Each voxel considered four populations of cells: tumour cells, capillary cells, normal cells and dead cells that may be present after irradiation. The number of capillary cells determined the state of oxygenation according to the vascular fraction *vf* (relative volume of capillary cells within a voxel). Since the amount of oxygen available may be heterogeneous in the cell microenvironment, a specific oxygen histogram was assigned to each voxel. The shape of the histogram depended on the vascular fraction of the voxel and was build according to the method described by Espinoza et al. [38]. Here, a series of six different oxygenation patterns was built, each one corresponding to a specific histogram, as depicted in Figure 1.

Once each voxel has been described according to these parameters, the proportions of cells evolve at each cell cycle time. At the microscopic scale, the model was based on the cellular division to handle tumour growth, according to classical phases of the cellular cycle:

- *G*_0_ phase, also called quiescence phase where the cell has left the active cycle and has stopped dividing.
- *G*_1_ phase, where the cell increases in size.
- *S* phase, or synthesis phase, where DNA is duplicated.
- *G*_2_ phase, preparing mitosis (synthesis of enzymes, etc.).
- *M* phase or mitosis, which is the last phase of the cell cycle, when division occurs.

Based on the work of Wille et al. [39], we speculated that the duration of the cell cycle *Tc* is 24 h. The initial cell cycle phase distribution was assumed to be 14 h in *G*_1_, 6 h in *S*, 3 h in *G*_2_ and 1 h in *M*. In general, there are two checkpoints in the cell cycle, one for controlling transition from *G*_1_ to *S* and another for controlling transition from *G*_2_ to *M.* The transition from *S* to *G*_2_ was assumed to be automatic. A cell that was in none of these stages was considered in the quiescence phase (*G*_0_).

The cell cycle was arbitrarily divided into intervals of 1 h, which is also the duration of each step in the simulation. A cell that entered phase *G*_1_ automatically passed through the first intervals but underwent a transition test at the end of the phase. In the case of success, the cell continued its cycle until the next checkpoint (at *G*_2_/*M*).

Checkpoint tests were given using a random variable that follows Bernoulli’s law [32]. The total number of cells at a checkpoint was thus governed by a random variable that follows the binomial law. Given the large number of cells in the population concerned, this binomial distribution was then approximated based on a normal distribution [32].

Concerning tumour response modelling, a cell in the cellular cycle can die, survive and proliferate.

**Table 1:** List of parameters of the simulated tumour model.

Parameter	Symbol	Value
**Cell density**	*µ*	10^6^/mm^3^ [40]
**Cell cycle time**	*Tc*	24 h
**Upper asymptote**	*C*	1 [41]
**Cell growth rate**	*B*	0.075 [41]
**Oxygen partial pressure**	${pO}_{2}$	Calculated from histograms
**Inflection point (the *pO*_2_ value at the point of maximum incline)**	M	26.3 [41]
**Capillary cell proliferation (doubling time)**	$t_{a}$	612 h [42]
**Radiosensitivity coefficient**	α	0.273 Gy^−1^ [43]
**Radiosensitivity coefficient**	β	0.045 Gy^−2^ [43]
**Dose**	d	2 Gy
**Maximum** OER **value**	m	3 [44]
**Oxygen partial tension at** $OER=\left(m+1\right)/2$	k	3 mmHg [45]
**Half-life of dead cell resorption**	tr	168 h [22]

### 2.2. Model Components

A series of conventional radiobiological processes [32,41] was used as the main components of the model.

- Proliferation of tumour cells: the probability of cell division depends on the current oxygenation status of the cell. Cell proliferation factor *PF* was calculated as follows:
  (1) $$PF=C.\exp\{-\exp\left[B.\left({pO}_{2}-M\right)\right]\}$$
  where *C* is the superior asymptote, *B* the growth rate and *M* represents the *pO_2_* value at the curve inflexion point.
- Angiogenesis: the presence of hypoxia may induce angiogenesis [46]. In this case the fraction of the capillary cells was multiplied by the factor ${PF}_{a}$:
  (2) $${PF}_{a}=\exp\left(\frac{\ln\left(2\right)}{t_{a}}\times time\right)$$
  where *t_a_* is the doubling time for capillary cells and time is the simulation time step (1 h).
- Cell survival after irradiation *SF*:
  (3) $$SF=\exp(-\frac{\alpha }{m}d.OER\left({pO}_{2}\right)-\frac{\beta }{m²}d²OER\left({pO}_{2}\right)²)$$
  Here, *α* and *β* are the radio-sensitivity coefficients, *m* is the maximum ratio, *d* is the dose and the OER is given by the following:
  (4) $$OER\left({pO}_{2}\right)=\frac{m{pO}_{2}+k}{{pO}_{2}+k}$$
  where *k* is the *pO*_2_ at half of the increase from 1 to *m.*
- Resorption of dead tumour cells: tumour cells that have died after irradiation will be resorbed after a few days. This was reflected by the fraction of resorption:
  (5) $$RF=1-\exp(-\frac{\ln\left(2\right)}{tr}\times time)$$
  Here, *tr* represents the half-life of dead cell resorption.
- Cell replacement: if the number of cells per voxel decreased after the resorption of dead cells, it was necessary to redistribute some new cells in the voxel to maintain cell density. For the sake of simplicity, resorbed cells were arbitrarily replaced by normal cells or capillary ones, depending on the oxygenation model.

### 2.3. Factors Influencing Tumour Response According to the 5 Rs

In this study, the developed model was obtained by adding the 5 Rs of radiobiology to the processes described above. In the literature, the values of these different parameters differ depending on the type of cancer. With regard to our study, the parameter values relate to the cancer “rhabdomyosarcoma”. Therefore, to determine each parameter, as well as to combine the model components, we relied on previous studies as explained below.

#### 2.3.1. Repair

The use of radiotherapy as a treatment modality exposes cells to ionizing radiation, which damages DNA. Cell survival after irradiation depends on the cell’s ability to repair itself and on the type of lesion. In fact, if DNA damage is irreparable, cells activate death programs [47]. In the case of normal tissue cells, if they can repair the damage, it can only be beneficial for the treatment result. In contrast, in the case of tumour cells, post-radiation survival allows tumour cells to proliferate [48]. The success of radiotherapy therefore depends on the extent of damage in the exposed tumour tissue [47].

In fractionated radiotherapy, by dividing the total dose into a set of fractions, repair produces increased cell survival by allowing cells to recover after the individual radiation dose and consequently proliferation between fractions. Generally, the linear-quadratic model is often used in radiobiology to describe the response to radiation [1]. It represents a mathematical formula linking cell survival and the radiation dose and depends on two parameters, *α* and *β*. The parameter *α* determines the initial slope of the cellular survival curve, and *β* represents the accumulation of sublethal damage.

Dose splitting allows time for healthy tissue to recover and repair itself between sessions, which is not the case for tumour cells as their repair system is disrupted by nature.

In this study, the density of surviving normal cells after irradiation is given by the following:

(6) $${SF}_{N}=(-\alpha d(1+d\frac{\beta }{\alpha }))$$

The density formula used in our model was inspired by the work presented by Joiner et al. [1]. Dead normal cells will be replaced as dead tumour cells as described above in “Cell replacement”. The control of DNA repair is closely linked to the redistribution of the cell in the cellular cycle [49]. In fact, checkpoints during the cell cycle ensure that DNA is intact before DNA replication and cell division.

#### 2.3.2. Redistribution in the Cell Cycle

During irradiation, cells can be anywhere in the cell cycle, at a given phase, among *G*_1_, *S*, *G*_2_ or *M*.

Cells are characterized by various radiosensitivities depending on their location in the cell cycle. Cells in phase *S* are known to be more radioresistant, while cells in other phases are relatively more sensitive to radiation [50]. Thus, the radiation dose delivered for an asynchronous population of cells will probably induce more deaths for cells that are in the sensitive phase. Consequently, cells that survive are those that were in resistant phases. As a consequence, the surviving population is partially synchronized [51].

Damage to DNA after irradiation will block cell progression at cell cycle checkpoints [52] causing cell accumulation at this point. This behaviour is expected to prevent cells from entering into the division phase with fatal damage [53]. Over time, surviving cells will continue to evolve in the cycle. If a second dose of radiation is delivered sometime later, some of these cells will have left the resistant phase and will be in a more sensitive phase, which will allow them to be killed more easily. In our study, we proposed to add a weighting coefficient described by Joiner et al. [1] to manage the sensitivity of cells to their phases before irradiation.

#### 2.3.3. Repopulation

Conventional fractionation consists of delivering a small dose (2 Gy) daily on a weekday during several weeks. This protocol allows normal tissues to repair potential radiation damage between two consecutive fractions. However, surviving tumour cells can also repopulate between fractions. Repopulation plays an important role in the outcome of radiotherapy, since surviving tumour cells can generate new cells that were not present at the beginning of the treatment.

Repopulation may have a significant role when the overall treatment time is long, as seen in classical fractionation protocols. However, there are strategies that can reduce the effect of repopulation through accelerated fractionation, which reduces the overall treatment time by delivering two fractions per day instead of one. In addition, an accelerated repopulation of malignant cells is often observed in a series of common cancers [54]. These types of tumours show accelerated repopulation after two or three weeks of treatment.

In this work, we chose to vary the doubling time *Tp*, defined as the time required for the tumour cell population to double without natural loss. As an example, during the first two weeks *Tp* is set to 1200 h and becomes 120 h until the end of treatment [1,55].

#### 2.3.4. Reoxygenation

Local oxygen tension plays an important role in the radiotherapy response [54]. Several studies showed that the poor prognosis of radiotherapy might be correlated with tumour hypoxia [56,57]. Thomlinson and Gray [58] have suggested that human lung cancer contains hypoxic areas, with necrotic areas closely related to the size of the tumour. In fact, when the tumour size is greater than 1 mm, cells located at a distance from the vessels have reduced access to oxygen, and the central part of the tumour becomes necrotic. On the contrary, when the diameter of tumours is less than 1 mm, they were found to be fully oxygenated [1]. In addition, the effect of ionizing radiation targeting DNA is stabilized in the presence of oxygen. In contrast, in hypoxic environments, tumour cells are two-to-three times more resistant than well-oxygenated cells. Moreover, when radiation is separated into multiple fractions delivered over weeks, cells that are relatively resistant due to hypoxia at the beginning of the treatment may become reoxygenated.

Reoxygenation is the phenomenon of hypoxic tumours becoming oxygenated again during radiotherapy treatment. Numerous studies have shown that the reoxygenation of tumours may occur only 24 to 72 h after irradiation [59]. Reoxygenation is caused by several mechanisms, such as reopening temporarily closed vessels or decreasing the respiration of fatally damaged cells. Other mechanisms need longer intervals to occur, such as cell death due to mitotic catastrophe or ischemia leading to tumour shrinkage and a decreased distance between capillaries and tumour cells that allows oxygen to reach the hypoxic zone.

In this work, oxygen levels varied in time and space. The vascular fraction *vf* varied during the treatment and changed from one voxel to another. In the model, *vf* depended on the proportion of capillary cells in voxels. Initially, every voxel contained 80% of tumour cells, and in the remaining 20%, 96.4% of normal cells and 3.6% of capillary cells were present [60]. This distribution would remain valid throughout the simulation, with dead cells replaced by capillary and normal cells in the proportions of 3.6% and 96.4%, respectively. The reoxygenation of malignant cells during radiotherapy can increase their radiosensitivity.

#### 2.3.5. Radiosensitivity

Tumours respond differently to radiation therapy. This variable response is correlated with the intrinsic radiosensitivity of cells. Radiosensitivity was added to the four Rs in 1989 by Steel et al. [61]. This new member of the Rs of radiobiology highlights the fact that there is an intrinsic radiosensitivity in different types of cells.

Tumour cell radiosensitivity is a significant prognostic factor for the overall response of tumours and radiotherapy outcomes. Some studies were interested in trying to determine and predict this inter-individual variability. In theory, it would be interesting to be able to determine the intrinsic radiosensitivity of tumour cells, as well as the radiosensitivity of healthy tissues, for each patient. Radiosensitivity, which is the relative susceptibility of cells to ionizing radiation, is usually indicated by the parameters of the linear-quadratic equations. For this reason, we used specific *α* and *β* parameters for each cell type, either healthy or tumoural.

### 2.4. Simulations

**Figure 2:**
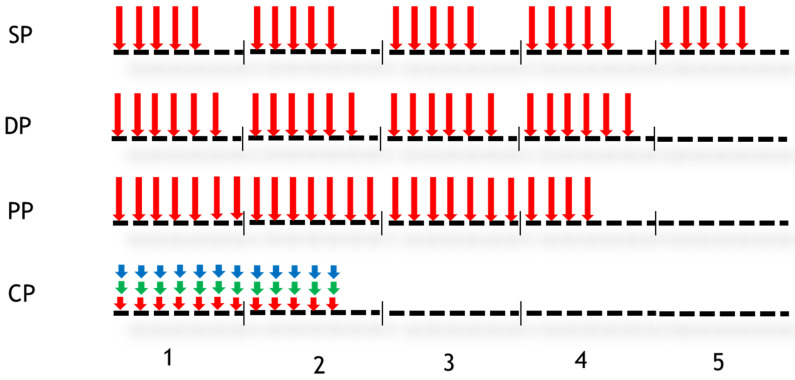
Different fractionation schedules. Vertical arrows indicate the days when the dose was performed. Four protocols were studied: Standard Protocol (SP), DAHANCA Protocol (DP), Personal Protocol (PP) and CHART Protocol (CP). In the case of CP, three doses were delivered per day (for other details, see the text).

In our study, four fractionation schemes were designed and simulated to quantify the impact on the tumour response to radiotherapy (Figure 2).

In Figure 2, vertical arrows indicate the days when the radiotherapy session takes place. The numbers 1 to 5 represent weeks. The first fractionation scheme studied is conventional or standard fractionation (SP: standard protocol), with a 2 Gy dose delivered 5 days per week for 5 weeks. The second fractionation scheme is sometimes referred to as DAHANCA (DP), in which a dose of 2 Gy is administered 6 days a week instead of 5, leading to an overall treatment time of 4 weeks instead of 5. The third fractionation scheme is a personal scheme (PP) in which a dose of 2 Gy is administered for 25 days without any interruption (weekends are included). The fourth fractionation scheme shown in Figure 2 is the CHART protocol. During this protocol, 36 fractions of 1.5 Gy over 12 consecutive days are administered. The treatment consisted of 3 fractions per day every 6 h. The tumour response was simulated for each fractionation scheme using simulated tumour and then PET images.

### 2.5. Data

In this study, artificially created tumours with simple geometries were used to demonstrate the main features of the model and to visualize the impact of the different biological effects. The virtual tumour had a diameter of approximately 3 cm in accordance with the work of Espinoza et al. ([60]).

Then, for validation, we used [18F] HX4 PET images from an image database generated by our previous study [62], which are considered a reliable tool for the detection of hypoxic tumour regions [37,63,64]. Data (images) from four mice with rhabdomyosarcoma tumours, with two control mice that received NaCl while two others received TH-302 for 4 days, have been used in this study. A [18F] HX4 PET image was acquired before treatment and a second one was acquired on day four of the treatment. Irradiation was delivered on day 3. During the treatment, animals were exposed to modified oxygen concentrations. Two mice were treated under carbogen (95% oxygen, 5% CO_2_) and the other two were treated under reduced oxygen breathing (7% oxygen):

- Mouse 1: NaCl + 95% oxygen;
- Mouse 2: TH-302 + 95% oxygen;
- Mouse 3: NaCl + 7% oxygen;
- Mouse 4: TH-302 + 7% oxygen.

The model input data are PET images and a distribution of the partial pressure of oxygen. The model returns simulated PET images. Throughout this study, we hypothesized that the number of tumour cells is proportional to glucose consumption as described elsewhere [28,41].

To calculate the initial distribution of cells in each voxel, we searched for the voxel with the maximum intensity (*i_max_*). Then, we divided the values of each voxel by the max. Then, *µ_t_* = 80% of the tumour cells were assigned to each voxel, and in the remaining 20%, *µ_n_* = 96.4% were normal cells and *µ_c_* = 3.6% capillary cells.

In a voxel, we have 10^6^ cells/mm^3^ [60], and denoting *i_max_* as the maximum intensity among all the intensity values of the voxels, the initial number of tumour cells in each voxel is given by the following:

(7) $$N_{tum}=\frac{i_{vox}}{i_{max}}\times V_{vox}\times \mu_{t}\times 10^{6}$$

where *V_vox_* is the volume of a voxel and *i_vox_* is the intensity of the voxel.

Therefore, a new PET image can be generated during the simulation using the updated value of the cell number value to calculate the intensities in this new image.

### 2.6. Tumour Control Probability (TCP)

TCP (Tumour Control Probability) is a model that predicts and quantifies the biologic radiation response of tumours, the survival. The most used model is based on Poisson statistics describing the distribution of the surviving tumour cells. In general, the LQ model is used for calculating cell survival and it allows for the quantification of TCP for tumours.

Here, a TCP curve was generated by simulating the tumour response to radiotherapy. We consider a tumour controlled if all tumour cells were killed.

## 3. Results

**Figure 3:**
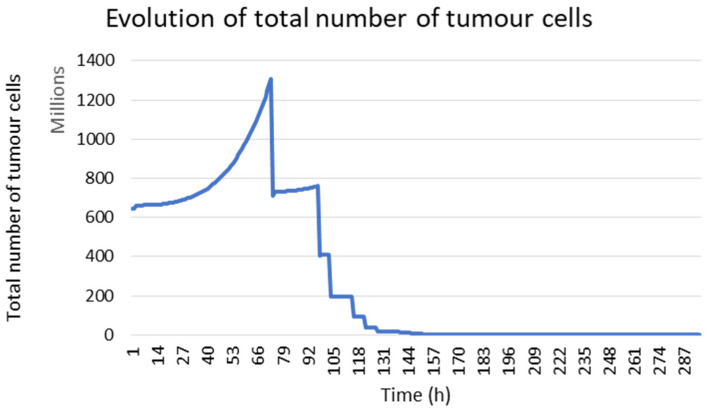
Evolution of the tumour cell number during the treatment in the case of mouse 1.

The presented model allows us to simulate the tumour response under radiotherapy. In Figure 3, the temporal evolution of the total number of tumour cells is presented. During the first 72 h, the increase in the number of tumour cells was due to the absence of radiotherapy. After this initial phase, the effect of radiotherapy becomes visible. A daily decrease in tumour cells followed by a recovery was observed due to the repopulation of tumour cells between two radiations.

**Figure 4:**
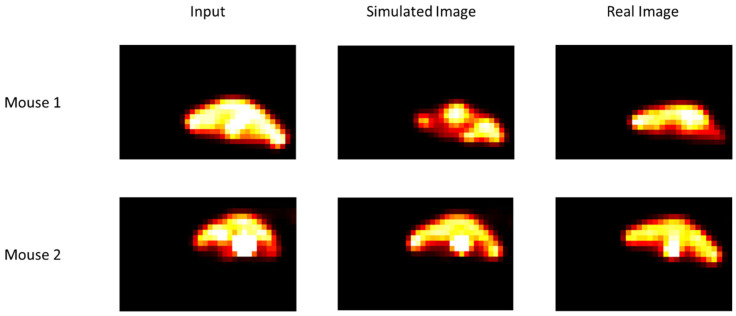
Comparison of simulated and real images for two mice under carbogen (95% oxygen, 5% CO_2_). Input images were acquired before the beginning of radiotherapy. Simulated and real images were obtained after a single irradiation (for other details, see the text).

**Figure 5:**
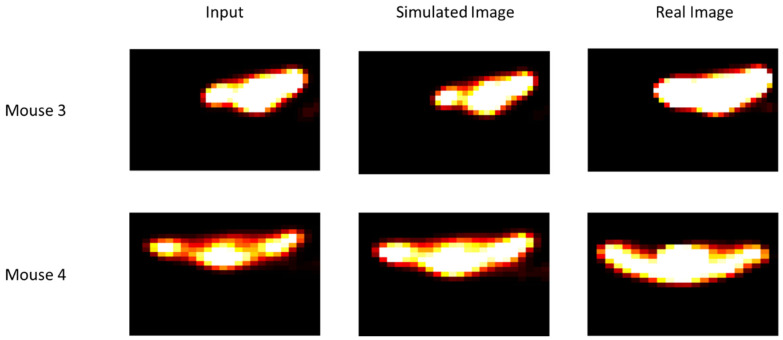
Comparison of simulated and real images for two mice under breathing 7% conditions. Input images were acquired before the beginning of radiotherapy. Simulated and real images were obtained after a single irradiation (for other details see text).

In Figure 4, tumour growth images are presented. Qualitatively, the visual comparison between real and simulated images shows that there is real similarity between them. The simulated image was obtained from the real images acquired before the beginning of treatment. Mice were under carbogen. Although these images were obtained after a single irradiation, we can see the effectiveness of radiotherapy, which is not the case in Figure 5.

In Figure 5, it can be noted that there is a certain resistance of the cells, which is quite logical because the mice were under limited oxygenation. In the case of mice under breathing 7% conditions, we imposed, at the beginning of the simulation, a hypoxic histogram to reproduce the conditions under which mice were treated. In addition, in the other case (under carbogen), we imposed a well-oxygenated histogram.

In the case of mouse number 3, the effect of radiotherapy is not visible. In the case of mouse 4, we can see a slight change. This result is also visible in the case of mouse 4, despite treatment with TH-302, an anti-tumour agent that is activated in a hypoxic environment.

### 3.1. Repair

**Figure 6:**
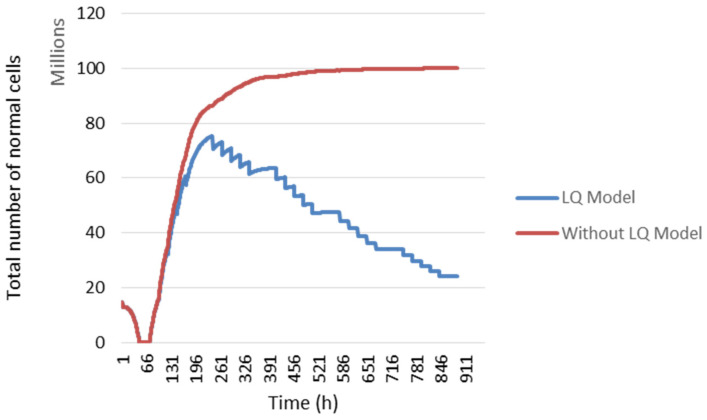
The evolution of normal cell numbers during the treatment. Comparison of the evolution using the LQ Model (blue curve) and without it (red curve) (for other details, see the text).

In our study, we modelled the response of normal cells to radiotherapy with the linear quadratic model. In Figure 6, the temporal evolution of normal cells is presented. The blue curve represents the evolution of normal cells using the linear quadratic model, which is not the case in the red curve.

During the first 72 h, the decrease in the total number of normal cells is due to the absence of radiotherapy. In fact, when radiotherapy is not administrated, the number of tumour cells per voxel increased. Since there is a fixed cell density in a voxel, tumour cells continued to proliferate at the expense of other cell types. Then, when radiotherapy was administered, the number of dead tumour cells following irradiation gave way to other cell types. Therefore, the number of normal cells increased after 72 h. Then, a slight change in this number was noted between two irradiations caused by cellular sensitivity to irradiation and cellular repopulation.

### 3.2. Redistribution in the Cell Cycle

**Figure 7:**
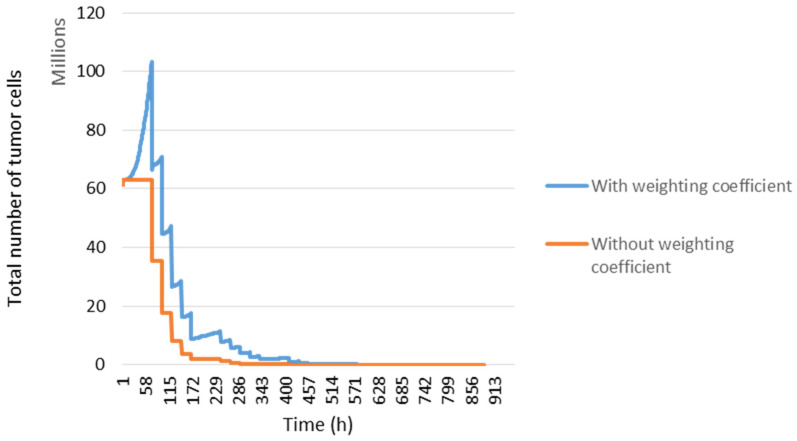
Tumour response and cell distribution in the cell cycle. The evolution of the number of tumour cells took into account the distribution in the cell cycle (blue curve) and without (orange curve).

The distribution of cells in the cell cycle affects the outcome of radiation therapy. A weighting coefficient was added to manage the sensitivity of the cells according to their location before irradiation (blue curve Figure 7), which is not the case in the orange curve.

**Figure 8:**
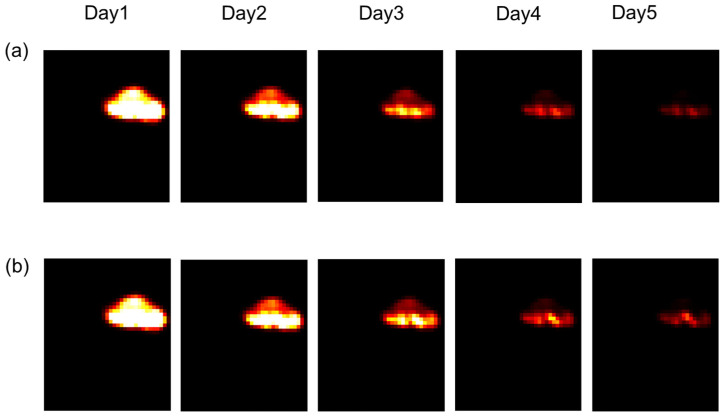
Tumour response in mice in the presence or absence of variations in radiosensitivity during the cell cycle. (**a**): Weighting coefficients varied, and in (**b**), coefficients were constant (for other details, see the text).

According to the work of Joiner et al. [1], we chose to modify the parameters during each irradiation. The aim was to see the impact of this variation on the result of the irradiation after one week of treatment. We observed in Figure 8 that from the third day, there was a difference between the irradiation administered with variations in the parameters (Figure 8a) and without variation (Figure 8b). It was deduced that variation of the parameters allows for the more rapid control of the tumour.

**Figure 9:**
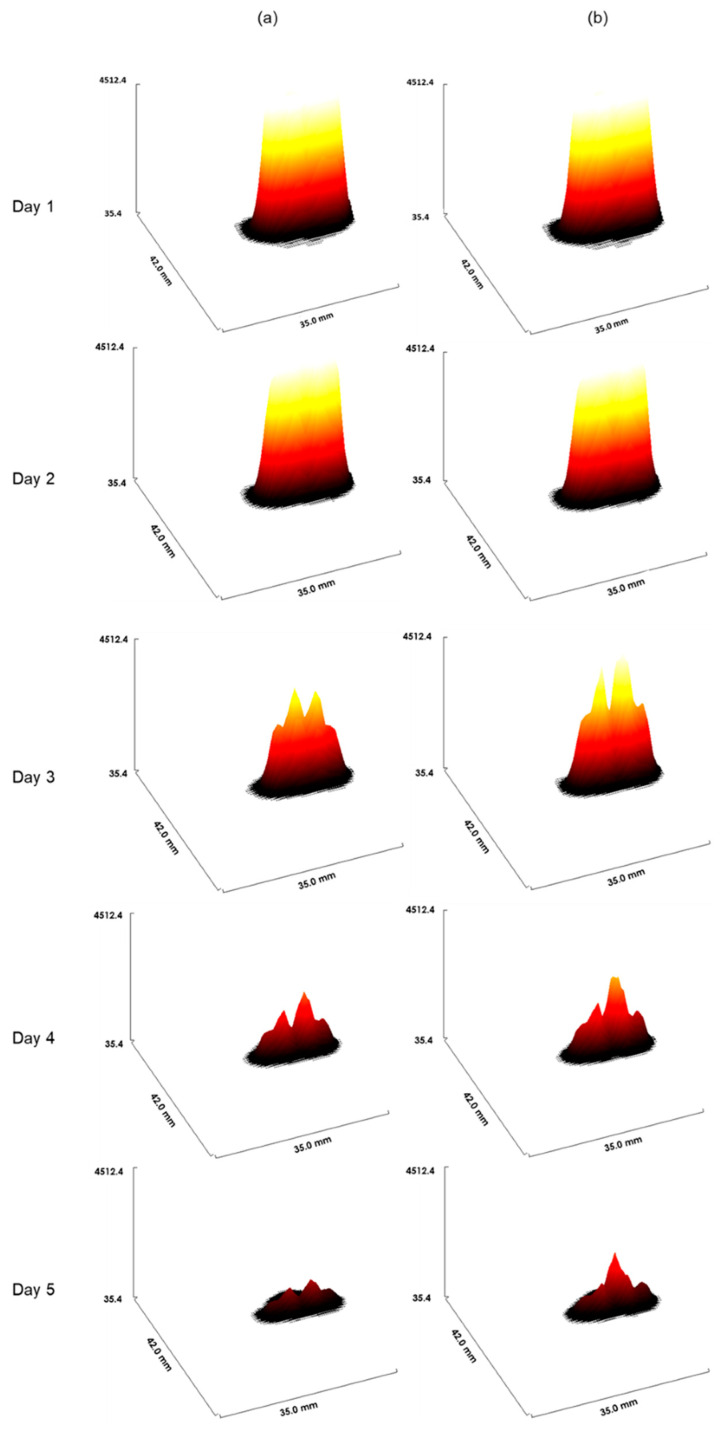
The interactive 3D surface plots displayed a three-dimensional graph of the intensities of voxels. (**a**): Parameters of radiosensitivity were varied with every irradiation. Of note, for each irradiation per day, a new coefficient was used, and in (**b**), parameters were fixed (for other details, see the text).

Figure 9 confirms the difference seen in Figure 8. In Figure 9a, parameters were varied during every irradiation (for each irradiation per day, we have a new coefficient), and in Figure 9b, the value of these parameters was constant during the treatment. In this figure, we have drawn an interactive 3D surface plot. These plots display a three-dimensional graph of the intensities of voxels in the image. In our study, we considered the closer the voxel intensity is to the black colour, the more we controlled the tumour. Indeed, the darkest areas are the areas where there is more oxygen. Thus, fewer tumour cells are in these areas.

In Table 2, a percentage of cells in each phase of the cell cycle has been shown. It is observed that the percentage of cells in *G*_1_ after 24 h increases slightly and remains stable in phase *M*. On the other hand, the percentage of cells in phase *S* decreased but it increased in phase *G*. This observed evolution corresponds to experimental data presented in the study [65].

**Figure 10:**
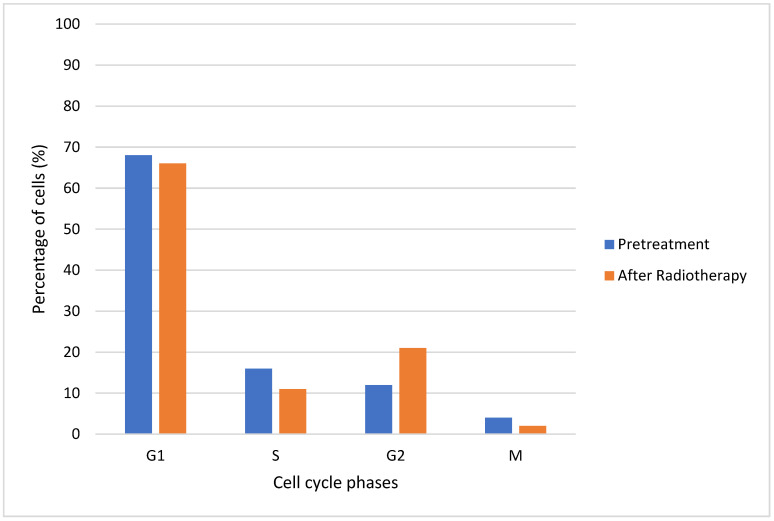
Percentages of cells in each cell cycle phase (before and after radiotherapy).

In Figure 10, a percentage of cells in each cell cycle phase before and after radiation therapy is shown. It was observed that there was a decrease in the percentage of cells after radiotherapy in phases *G*_1_, *S* and *M*, which was not the case for phase *G*_2_. Compared to the initial condition, for a dose of 2 Gy, there was a large increase in cells in phase *G*_2_ after radiotherapy. The blockage of cells in *G*_2_ following radiation exposure can be attributed to the activation of cellular repair mechanisms. As a result, the percentage of cells in *M* phase decreased at the same time. The percentages of *G*_1_ and *S* cells also declined following irradiation [65].

**Figure 11:**
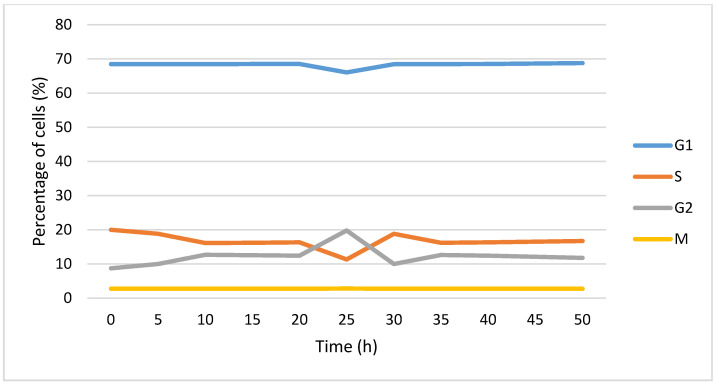
Evolution of percentage of cells in each cell cycle phase over time. The cells were irradiated at t = 20 h.

In Figure 11, evolution of the cell cycle phases over time is presented. The irradiation takes place at t = 20 h. It is observed that following radiotherapy, the percentage of cells in *G*_1_ and *S* decreases and increases in *G*_2_. After this disturbance (t = 30 h), the percentage in *G*_1_ and *S* increases and that in *G*_2_ decreases.

### 3.3. Repopulation

**Figure 12:**
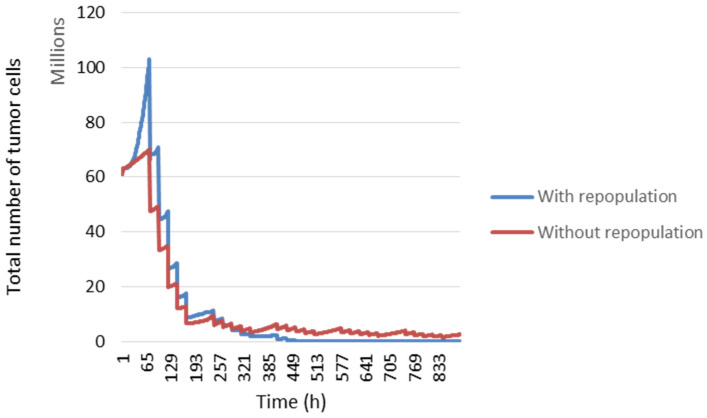
Evolution of number of tumour cells with or without tumour repopulation over time.

During the first two weeks of radiotherapy, *Tp* will be equal to 1200 h and then change to 120 h until the end of the treatment. In Figure 12, the blue curve represents tumour development considering repopulation, but in the red curve, cell repopulation is not considered.

### 3.4. Reoxygenation

Here, we considered the spatiotemporal evolution of oxygen during radiotherapy treatment.

**Figure 13:**
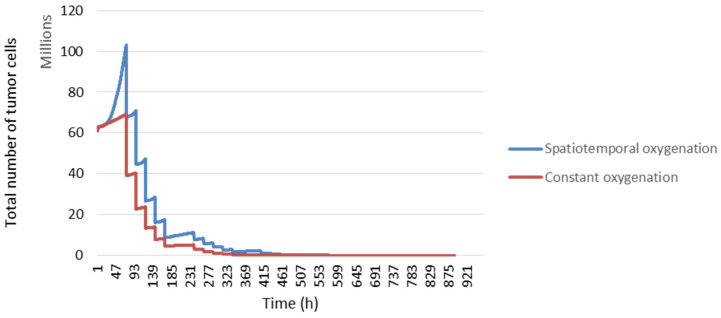
The response of tumour cells according to tumour oxygenation.

In Figure 13, we compared the tumour response with the spatiotemporal evolution of oxygenation (blue curve) and constant oxygenation (red curve). We can see that our model is sensitive to changes in the rate of the partial pressure of oxygen.

### 3.5. Radiosensitivity

In this study, the radiosensitivity parameters were varied to see their impact on the tumour response. The values of these parameters have been reported by three different studies. At the start, we used linear quadratic model parameters *α* = 0.339 and *β* = 0.067, which gave a *α*/*β* ratio equal to 5.06 Gy [66]. Second, the tumour response was simulated with *α* = 0.04 and *β* = 0.0089 (ratio *α*/*β* = 4.94 Gy) [32]. Finally, we worked with an *α*/*β* ratio = 6.06 Gy [1].

**Figure 14:**
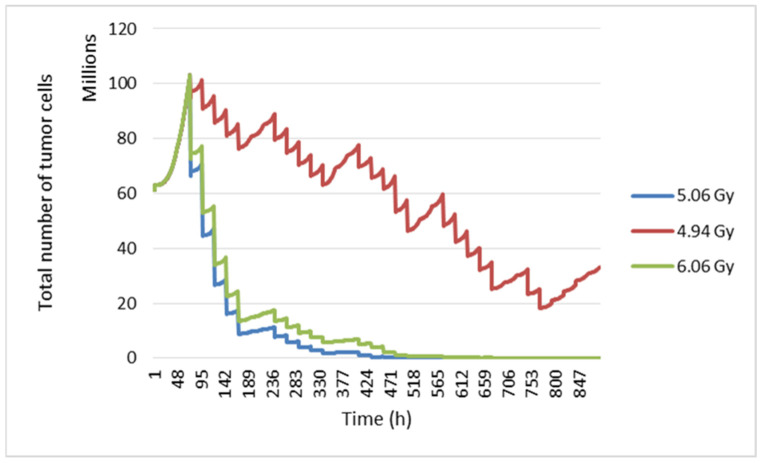
Evolution of tumour cell numbers over time according to the *α*/*β* ratio.

By modifying the *α* and *β* radiosensitivity parameters, a different tumour response is obtained (Figure 14). This shows that our model considers intrinsic radiosensitivity.

To demonstrate the main features of the model and visualize the impact of different fractionation patterns on tumour response, an artificially created virtual tumour with simple geometries was used in this study as a first step. Then, to validate the results obtained with the simulated tumour, the tumour response was simulated using a real image of a rhabdomyosarcoma tumour.

### 3.6. Tumour Response to Different Fractionation Schemes

#### 3.6.1. Simulated Tumour Response

**Figure 15:**
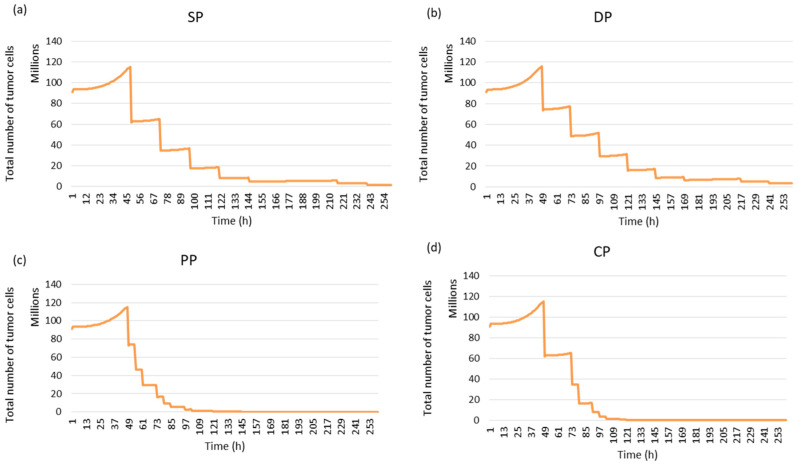
Tumour response to radiotherapy for each fractionation protocol using simulated images: (**a**) Standard Protocol: SP, (**b**) DAHANCA Protocol: DP, (**c**) Personal Protocol: PP and (**d**) CHART Protocol: CP.

In this study, four fractionation schemes were simulated. The tumour response to radiotherapy was then studied for each treatment regimen. It can be seen in Figure 15 that the personal protocol allows the tumour to be controlled more quickly than the other protocols. It is closely followed by the CHART protocol.

**Figure 16:**
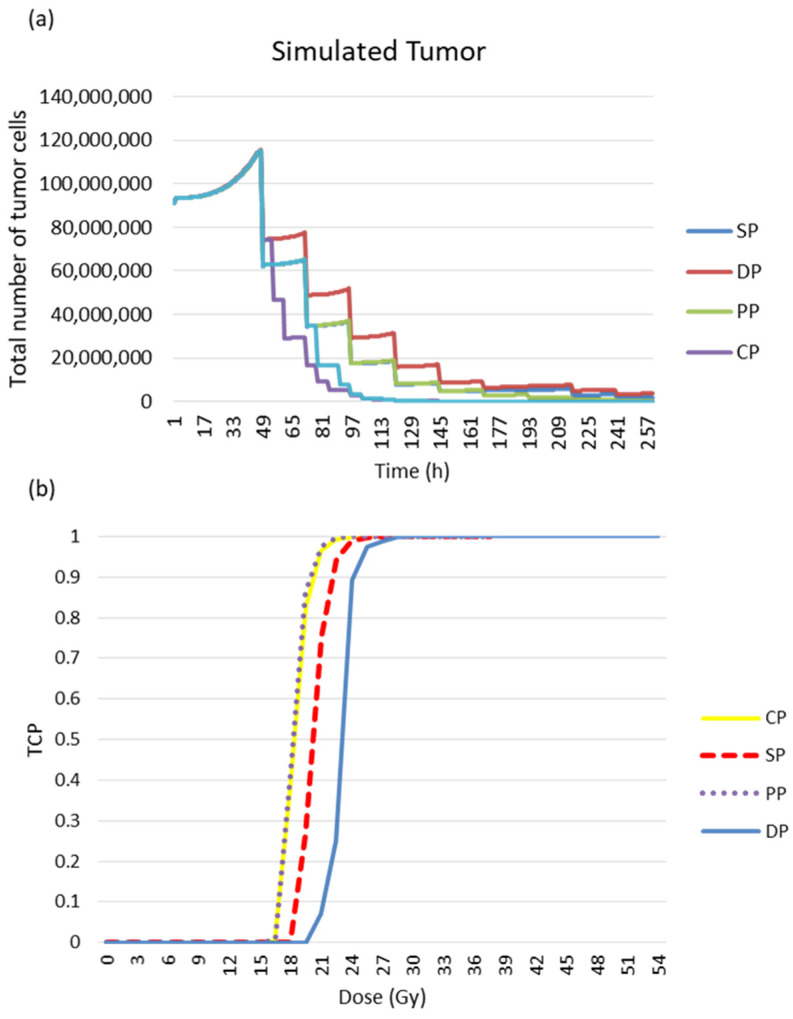
Tumour response to radiotherapy. (**a**) Evolution of tumour cell numbers over time, and (**b**) the TCP curve for the simulated tumour. The TCD50 values (in Gy) of the TCP curves are 17.7, 17.9, 20.1 and 23.4 for PP, CP, SP and DP, respectively.

The following figure confirms the result observed in Figure 15. The simulation of the response of the simulated tumour to radiotherapy leads to different responses for the different treatment regimens (see Figure 16a). The PP and CP protocols provide faster tumour control than the conventional regimen and the DAHANCA protocol.

The tumour response was quantified in terms of cell survival and TCP curves (Figure 16b). The TCD_50_ (the dose required to control 50% of tumours locally) was calculated. The TCD_50_ values (in Gy) of the TCP curves are 17.7, 17.9, 20.1 and 23.4 for PP, CP, SD and DP, respectively.

#### 3.6.2. Tumour Response to Radiotherapy

**Figure 17:**
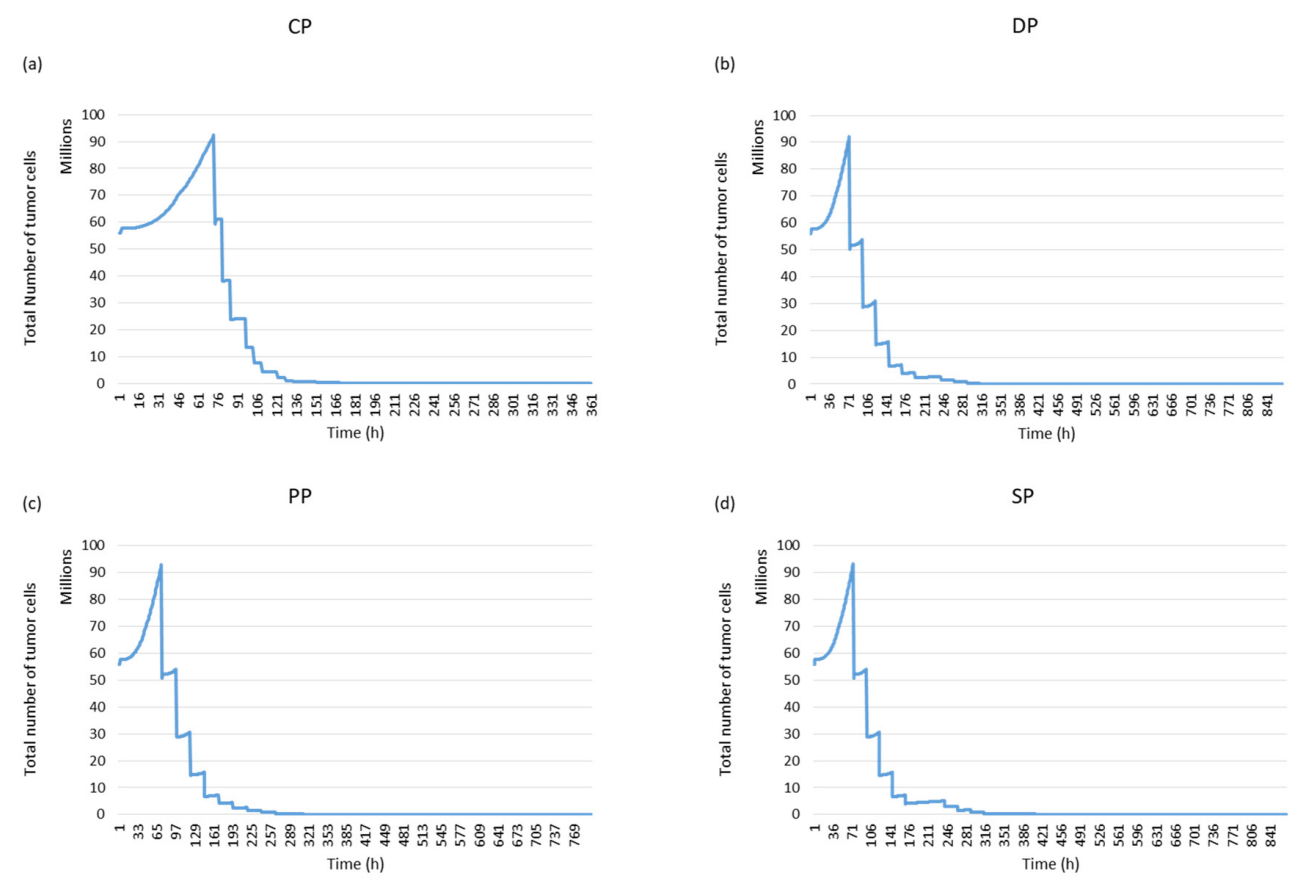
The evolution of tumour cell numbers over time for a rhabdomyosarcoma tumour based on each protocol: Standard Protocol (SP), DAHANCA Protocol (DP), Personal Protocol (PP) and CHART Protocol (CP).

In this study, we used [18F] HX4 PET images of mice with rhabdomyosarcoma as input for our simulation model. Figure 17 demonstrates that with the CHART protocol, we have better local control of the tumour.

**Figure 18:**
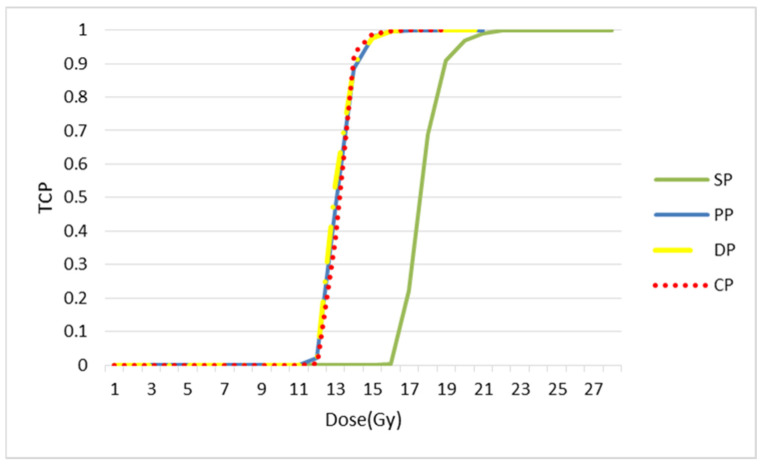
TCP curves for each splitting protocol. The values of TCD50 are 13.23, 13.24, 13.72 and 18.18, respectively for CP, PP, DP and SP.

In Figure 18, the TCP curve is plotted. Three fractionation protocols have been brought together: CHART, staff and DAHANCA. According to this curve, these three protocols are more efficient in terms of local tumour control than the classic or standard model. As with the simulated images, the values of TCD50 were calculated. The values were found to be 13.23, 13.24, 13.72 and 18.18, respectively for CP, PP, DP and SP.

**Figure 19:**
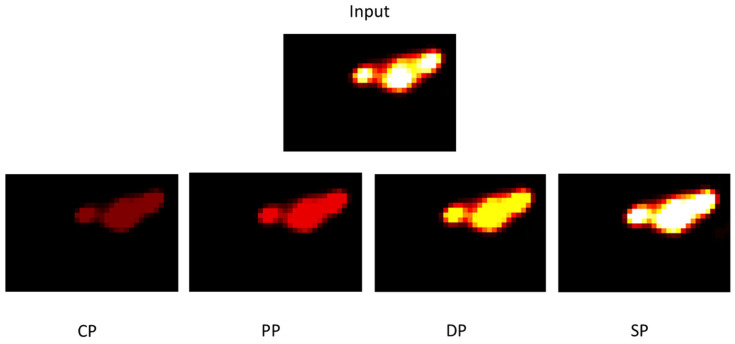
Tumour images corresponding to the tumour on the seventh day after the start of treatment for each protocol.

Figure 19 illustrates tumour development for each fractionation scheme. The images obtained on the seventh day after the start of treatment show that the CHART protocol has better tumour control, especially compared to the conventional protocol.

## 4. Discussion

In the context of the numerical simulation of tumour growth and response to radiotherapy, we proposed to combine two series of biological processes. The first set includes the main processes often studied independently so far, such as tumour cell proliferation, angiogenesis, cell survival after irradiation, resorption of dead tumour cells and cell replacement. The second set includes the main concepts of what is referred to as the five Rs of radiobiology. These five Rs are cell cycle redistribution, reoxygenation, intrinsic radiosensitivity, cell repopulation and repair. This is the first approach where these concepts are integrated into a unified environment, using both the microscopic cell cycle scale and the macroscopic population scale. To our knowledge, no other study on the modelling of the tumour response to radiotherapy considered these processes altogether. Most previous studies indeed simulated these processes separately [67], for example without considering reoxygenation despite its importance in the response to treatment. The approach presented here has several important advantages, such as versatility and the possibility of using it in different contexts with all kinds of input data, for example patient-specific biological data.

More specifically, we have shown that our approach responds correctly to the modification of input parameters, through application to a virtual cell population. We have also shown that it is possible to quantify the impact of fractionation on the response, by studying a theoretical population. An example of a practical evaluation is also proposed, using, as input data, a set of preclinical [18F] HX4 PET images acquired under different oxygen conditions. Images obtained after simulation were consistent with the real post-irradiation images. Moreover, our model has been used to test [18F] FDG PET images (non-hypoxia tracer) of patients with rectal cancer, and the results are promising (data not shown).

In this study, we investigated the impact of four different fractionation schemes on the evolution of a cell population. The objective was to find the protocol that allowed for better control of the tumour while limiting healthy tissue damage. The results showed that the reduction in treatment time has a considerable effect on local tumour control. In the cases of simulated and real tumours, the CHART protocol showed better tumour control, especially when compared with the conventional protocol. In the case of rectal cancer, studies have shown that the CHART protocol, as a preoperative treatment for rectal cancer, is feasible and appears to be associated with low acute and late toxicity. In the study of S. Brooks et al. [68], the authors found that local control was encouraging and therefore justified a more in-depth evaluation of this protocol. Thus, they proposed the incorporation of chemotherapy, which could also be considered as an option of investigation in the future.

Images used in this work were images of mice treated with NaCl or TH-302. TH-302 is an investigational hypoxia-activated prodrug used to treat cancer. TH-302 is activated only in hypoxic regions where the oxygen level is very low. It was developed to target the hypoxic zones existing in tumours. Generally, it is used combination with chemotherapy and shows rather satisfactory results. Recently, in the study of Peeters et al. [62], the authors studied the efficacy of the combination treatment of TH-302 and radiotherapy. In perspective, it would be better to model the TH-302 effect on the tumour and combine its effect with that of radiotherapy and then compare the simulated images with the images of the mice obtained.

As future work, it will be interesting to simulate different dose-painting strategies targeting hypoxic regions in tumours. Indeed, a non-uniform response can be observed within the tumour due to the heterogeneity of oxygenation. This point is consistent with the fact that hypoxic cells are more radioresistant than well-oxygenated ones, which can negatively affect the radiotherapy result. Simulation could provide insights about the non-uniform dose distribution required to destroy an equal proportion of cells within a heterogeneous tumour. In recent work, research demonstrated that radiotherapy induces an immune response. During radiotherapy, some immunosuppressive barriers might increase. This process can lead to tumour resistance. Boustani et al. [69] reviewed the effect of fractionation and the dose on the anti-tumour immune response. They propose a sixth R to the five Rs of radiobiology. This sixth one is reactivation of the immune system. Therefore, on the horizon, it would be interesting to find a way to model this sixth R.

## 5. Conclusions

For radiotherapy, the treatment outcome is determined by several factors. Some factors diminish the effect of radiation therapy, for example, the repopulation of tumour cells and repair from sublethal damage. Some factors increase the local control of the cancer, such as processes of reoxygenation and the redistribution of tumour cell into more sensitive phases of the cell cycle. The impact of the individual factor varies across different tissues, but it is important to consider all of these factors when developing a successful radiotherapy model. In the presented work, we tried to develop a model considering all five Rs of radiobiology. The model developed could be expanded in the future to include patient biological data for a truly specific clinical tool.

## Figures and Tables

**Table 2:** Evolution of phases (without treatment).

Time (h)	Percentage of Cells in:			
	*G*_1_	*S*	*G*_2_	*M*
0	68.44	20.04	8.77	2.75
24	68.58	16.44	12.22	2.75

## Data Availability

Data will be provided if required.
